# Exercise‐induced vasculitis with histological and genetic evidence of complement involvement

**DOI:** 10.1111/ddg.70240

**Published:** 2026-04-06

**Authors:** Massimo Cugno, Paolo Calzari, Alessandra Di Benedetto, Francesca Laura Boggio, Chiara Moltrasio, Luigi Porcaro, Angelo Valerio Marzano

**Affiliations:** ^1^ Internal Medicine Fondazione IRCCS Ca' Granda Ospedale Maggiore Policlinico Milan Italy; ^2^ Department of Pathophysiology and Transplantation Università degli Studi di Milano Milan Italy; ^3^ Dermatology Unit Fondazione IRCCS Ca' Granda Ospedale Maggiore Policlinico Milan Italy; ^4^ Pathology Unit Fondazione IRCCS Ca' Granda Ospedale Maggiore Policlinico Milan Italy; ^5^ Medical Genetics Laboratory Fondazione IRCCS Ca' Granda Ospedale Maggiore Policlinico Milan Italy

Dear Editors,

Exercise‐induced vasculitis (EIV), also referred to as exercise‐induced purpura, is a benign, small‐vessel cutaneous vasculitis triggered by prolonged physical activity, particularly walking in warm climates.^1^ It typically affects individuals without underlying systemic disease and has been reported in various settings such as marathons, amusement parks, and hiking trails. Despite its distinctive features, data on the epidemiology of EIV are currently lacking. Due to its occurrence in various recreational and sporting contexts, EIV has been described under multiple names, including Disney rash, golfer's vasculitis, hiker's purpura, jogger's petechiae, sport‐induced vasculitis, and marathon runner's vasculitis. Warm weather and prolonged exertion are key contributors to its onset. Clinically, EIV presents with typical skin manifestations such as palpable purpura and short‐lived hemorrhagic macules accompanied by burning, mild pain and pruritus, localized to the lower extremities, in the absence of systemic involvement.^1^, ^2^ As for all the other cutaneous vasculitides, the diagnosis of EIV is based on the combination of clinical aspects, histopathological features and direct immunofluorescence findings. The timing of biopsy is extremely important, as emphasized in a recent position paper.^3^ If feasible, conducting two biopsies on recent vasculitic lesions (< 48 h) is recommended: one from a partially blanchable macule for direct immunofluorescence analysis, and another from a non‐necrotic purpuric papule for routine hematoxylin and eosin evaluation.^3^ Histopathological findings commonly include leukocytoclastic vasculitis, with deposition of C3 and IgM in 86.7% and 46.7% of cases, respectively.^1^ Nevertheless, the condition is frequently misdiagnosed due to overlapping features with other purpuric disorders such as capillaritis, environmentally induced purpura, or purpura signaling systemic disease and its relationship with the so‐called recurrent macular vasculitis in hypergammaglobulinemia is not completely defined. The latter is likely the same entity since the hypergammaglobulinemia can also be lacking.^2^, ^4^ Although the pathophysiology of EIV is not fully understood, it is postulated that physical exertion under warm conditions may facilitate the formation of circulating immune complexes and activate the complement cascade. This, coupled with muscle fatigue and venous stasis, may contribute to capillary damage and the resulting vasculitic changes. Management is generally symptomatic, as the condition tends to resolve spontaneously within 7–10 days.^1^ Treatment options include NSAIDs, topical corticosteroids, and leg elevation, although their efficacy remains uncertain. A better definition of pathogenic mechanisms and predisposing factors could be useful in developing targeted therapies.

We report the case of a 75‐year‐old woman who has experienced recurrent episodes of purpuric lesions on the lower legs since 2016. The lesions appeared consistently after long walks, particularly in mountainous areas, and were localized between the ankles and knees. The patient's medical history included ascending aortic aneurysm (surgically treated), retinal vein occlusion (managed with intravitreal anti‐VEGF therapy), hypertension, and polymyalgia rheumatica. In August 2024, following a mountain walk, she developed a new episode of purpuric lesions (Figure 1) and was evaluated at our center. A 4‐mm punch biopsy was performed. Histopathological examination showed leukocytoclastic vasculitis affecting small vessels, with fibrinoid necrosis, perivascular neutrophilic infiltrate, and leukocytoclasia. C3 deposition was observed at the vascular level, while no significant immunoglobulin deposits were detected (Figure 2). Circulating immunoglobulins were within the normal range. These findings supported a diagnosis of EIV in the absence of hypergammaglobulinemia. Topical corticosteroids were prescribed for 10 days, leading to complete remission of the lesions.

**FIGURE 1:**
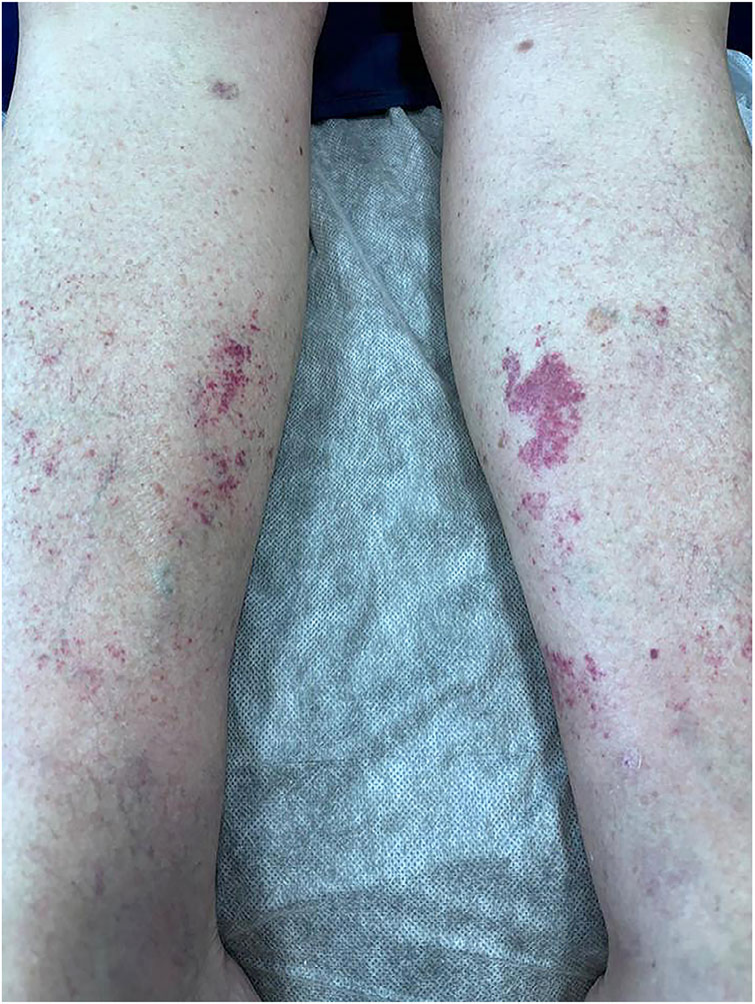
Macular purpuric lesions on the lower legs.

**FIGURE 2:**
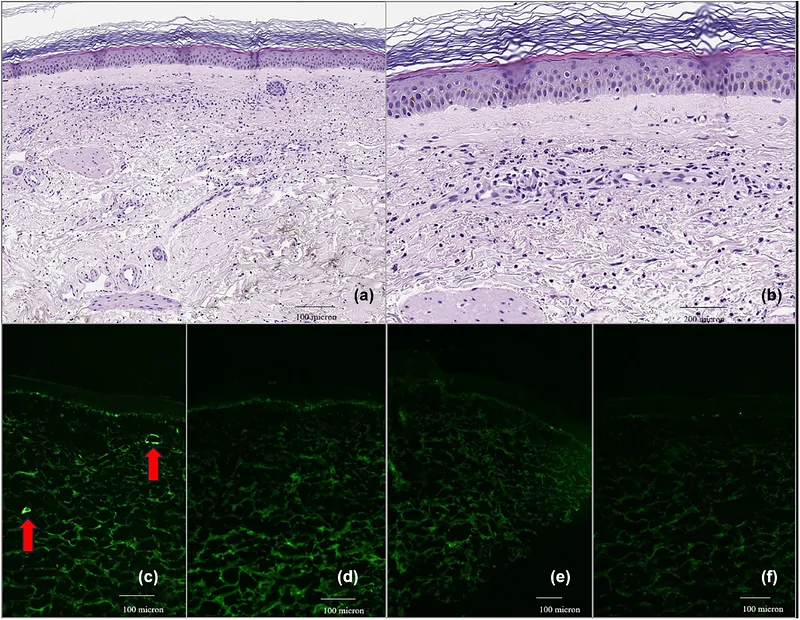
(a) Hematoxylin and eosin, 20x (scale bar, 100 µm): Mild perivascular inflammatory infiltrate composed of mononuclear and neutrophilic cells around small vessels in the superficial dermis; (b) Hematoxylin and eosin, 40x (scale bar, 200 µm: Lymphocytes and polymorphonucleated elements along with nuclear debris in the perivascular space with mild edema. (c) Direct immunofluorescence, 20x (scale bar, 100 µm): Granular C3 deposits within superficial dermal small vessels (red arrows). Direct immunofluorescence, 20x: No deposit where evident with (d) IgG, (e) IgA and (f) IgM.

To explore possible genetic predispositions to the vascular C3 deposition, complement related genes were evaluated by next‐generation sequencing (NGS) with a panel comprising *CFH* (NM_000186.4), *MCP/CD46* (NM_002389.4), *CFI* (NM_000204.5), *C3* (NM_000064.4), *CFB* (NM_001710.6), *THBD* (NM_000361.3), *DGKE* (NM_003647.3), *CFHR1* (NM_002113.3), *CFHR3* (NM_021023.6), *CFHR5* (NM_030787.4) and multiplex ligation‐dependent probe amplification (MLPA) to identify *CFHR3/CFHR1* copy number and macro rearrangements such as *CFH/CFH*‐Related hybrid genes. The analyses revealed only a heterozygous *CFHR3/CFHR1* deletion. It has been shown that *CFHR3/CFHR1* deletion is associated with an excessive complement activation leading to a thrombotic microangiopathy known as atypical hemolytic uremic syndrome,^5^ and with an increased susceptibility to systemic lupus erythematosus.^6^ Our patient has normal kidney function and has never had any kidney disease. However, the susceptibility to complement activation, related to *CFHR3/CFHR1* deletion, may facilitate the deposition of complement components in skin microcirculation during exercise because it is known that exercise is associated with complement activation leading to C3 consumption.^7^ Our observation could provide the rationale for proposing studies aimed at evaluating whether *CFHR3/CFHR1* deletion is also associated with increased susceptibility to EIV, supporting the role of complement activation in the pathophysiology of this vasculitis.

## FUNDING

This work was partially supported by the Italian Ministry of Health (Ricerca Corrente) of Fondazione IRCCS Ca’ Granda Ospedale Maggiore Policlinico, Milan (Italy).

## CONFLICT OF INTEREST STATEMENT

None.
